# Metabolomic and transcriptomic analyses revealed potential mechanisms of *Anchusa italica* Retz. in alleviating cerebral ischemia–reperfusion injury via Wnt/β-catenin pathway modulation

**DOI:** 10.1007/s13659-024-00495-3

**Published:** 2025-01-08

**Authors:** Wenta Tan, Shuo Fu, Yufei Wang, Bojun Hu, Guiquan Ding, Li Zhang, Wen Zhang, Guanhua Du, Junke Song

**Affiliations:** 1 Institute of Materia Medica, Chinese Academy of Medical Sciences and Peking Union Medical College, Beijing, 100050 China; 2 School of Pharmacy, Guangdong Pharmaceutical University, Guangzhou, 510006 China; 3 Qingdao Center for Disease Control and Prevention, Qingdao, 266000 China; 4 School of Pharmacy, Henan University, Kaifeng, 475004 China; 5 Prescription Laboratory of Xinjiang Traditional Uyghur Medicine, Xinjiang Institute of Traditional Uyghur Medicine, Urumqi, 830000 China; 6 Xinjiang Key Laboratory of Uygur Medical Research, Xinjiang Institute of Materia Medica, Urumqi, 830004 China

**Keywords:** *Anchusa italica* Retz., Cerebral ischemia–reperfusion injury, Transcriptomics, Metabolomics, Wnt/β-catenin signaling pathway

## Abstract

*Anchusa italica* Retz. (AIR), a traditional herbal remedy, is commonly applied in managing heart and brain disorders. However, its specific function and mechanism in acute cerebral ischemia–reperfusion injury (CIRI) are not fully understood. This research focused on the interventional effects and potential mechanisms of AIR extract (AIRE) in a rat model of CIRI. The model was established using the filament occlusion method, which involved blocking the middle cerebral artery for 1.5 h and then removing the filament to restore blood flow. Transcriptomic and metabolomic analyses were conducted to explore the molecular pathways and metabolites affected by AIRE. ATP level was measured using an ATP assay kit. Additionally, RT-qPCR and western blot tests were conducted to evaluate the influence of AIRE on the Wnt signaling pathway and mitochondrial function. Transcriptomic and metabolomic analyses indicated that AIRE regulated the Wnt signaling pathway in CIRI and modulated metabolites associated with mitochondrial energy metabolism, such as citrate and succinate. ATP assay result demonstrated that AIRE enhanced ATP production in CIRI. Further, RT-qPCR and western blot analyses revealed that AIRE activated the Wnt/β-catenin signaling pathway and corrected mitochondrial dysfunction. These results proposed that AIRE mitigated mitochondrial energy metabolism deficits in CIRI via the Wnt/β-catenin pathway. By restoring the balance of mitochondrial function and energy metabolism, AIRE might offer a potentially therapeutic strategy for addressing CIRI.

**Graphical Abstract:**

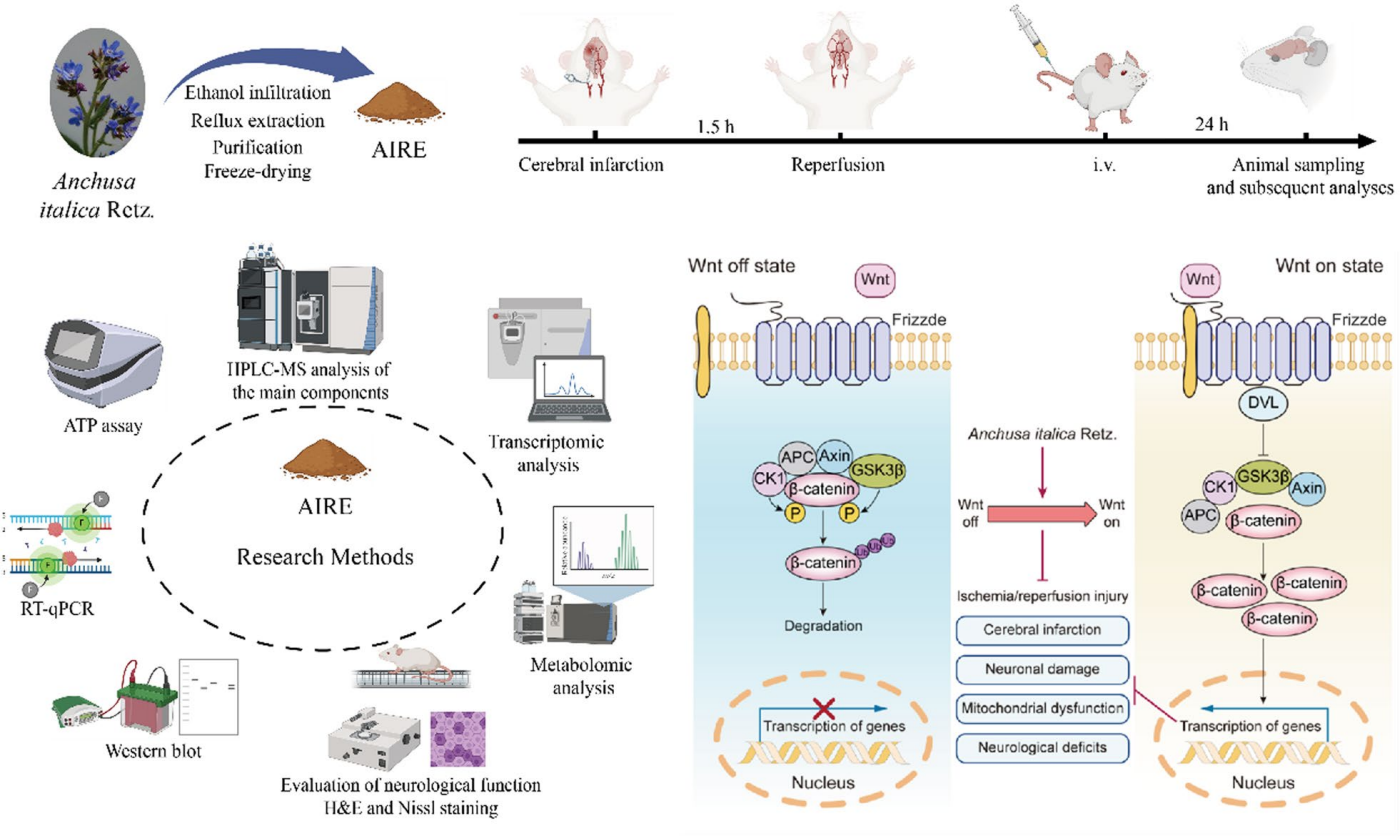

**Supplementary Information:**

The online version contains supplementary material available at 10.1007/s13659-024-00495-3.

## Introduction

Stroke has a substantial influence on public health because of its high rates of disability and mortality [1]. The incidence of stroke has increased considerably in recent years, especially among older adults [2, 3]. Ischemic stroke is the most prevalent form of stroke. During cerebral ischemia, inadequate blood supply to brain tissue results in an initial depletion of oxygen and nutrients, followed by a reversible loss of tissue function. If the ischemia persists, brain tissue begins to lose neurons and supporting structures, triggering a cascade of ischemic damage events [4].

Current treatment methods for ischemic stroke include thrombolysis and mechanical thrombectomy. These treatments aim to achieve blood reperfusion in the ischemic region, restoring blood and oxygen supply to brain tissues in time to salvage the potentially reversible ischemic penumbra and reduce further damage [5]. Unfortunately, achieving rapid reperfusion is limited by a strict therapeutic time window. Restoring blood flow to the ischemic region may lead to more severe brain tissue and nerve damage, known as cerebral ischemia–reperfusion injury (CIRI) [6]. Although clinical trials have shown that restoring blood perfusion after a prolonged period of ischemic stroke positively impacts the treatment of acute ischemic stroke, reperfusion-induced production of reactive oxygen species (ROS) often exceeds the antioxidant capacity of brain tissues [7]. This leads to a large accumulation of free radicals, disrupting neuronal homeostasis and resulting in inflammatory responses, oxidative stress, apoptosis, necrosis, and other pathological processes. Ultimately, this leads to cell death and tissue damage, posing more serious challenges for clinical treatment [8]. Thus, there is a critical need for effective medications to address CIRI damage.

Traditional Chinese Medicine (TCM) has a well-documented and extensive history of use in preventing and treating cerebral ischemia and ischemia–reperfusion injury. Currently, several Chinese patent medicines are widely utilized in clinical practice across China for managing these conditions. These include Naoxintong capsule [9], Xuesaitong injection [10], Xueshuantong injection [11], Shuxuening injection [12], Diterpene ginkgolides meglumine injection [13], Danhong injection [14], Salvianolic acids for injection [15], and Shuxuetong injection [16], et al. These medicines typically contain extracts from one or more common traditional Chinese herbs or animal-derived medicinal substances. For example, Naoxintong capsule is made from a combination of more than 10 traditional Chinese medicines, including *Astragali Radix*, *Paeoniae Radix* Rubra., and *Salvia miltiorrhiza*, et al. Xuesaitong and Xueshuantong injections primarily contain *Panax notoginseng* saponins. Shuxuening injection is derived from *Ginkgo biloba* leaf extract. Diterpene ginkgolides meglumine injection is composed mainly of Ginkgolides A, B, and K, all derived from *Ginkgo biloba* leaf extract. Danhong injection is based on extracts from *Salvia miltiorrhiza* and *Carthamus tinctorius*. Salvianolic acids for injection are enriched with phenolic acid compounds from *Salvia miltiorrhiza*. Shuxuetong injection contains extracts from leeches and earthworms, two common animal-derived traditional medicines. To conclude, most of the widely used drugs for the treatment of ischemia and ischemia–reperfusion injury in clinical practice are Chinese patent medicines developed based on TCM using modern pharmaceutical preparation techniques.

*Anchusa italica* Retz. (AIR), a medicinal herb widely distributed in China, Iran, Italy, etc., has a long history of usage in traditional remedies for treating a range of ailments. Chemical analyses have uncovered that AIR extract (AIRE) was rich in bioactive compounds [17–21], contributing valuable properties to its medicinal profile. The earlier research of our lab investigated the effects of AIRE on myocardial ischemia/reperfusion injury in rats, showing that AIRE dose-dependently reduced myocardial infarction index, decreased myocardial enzyme leakage, and improved cardiac function [22]. Mechanistic studies revealed that AIRE reduced inflammatory cytokines (IL-1β, IL-6, TNF-α) and modulated apoptotic proteins by increasing anti-apoptotic Bcl-2 expression and decreasing pro-apoptotic Bax expression, indicating significant cardioprotective effects through anti-inflammatory and anti-apoptotic pathways. Subsequent research from our lab demonstrated that AIRE enhanced the survival rate in post-myocardial infarction mice, reduced infarct size, and decreased inflammatory factors such as TNF-α, IL-1β, and IL-6, while downregulating pro-apoptotic markers like cleaved caspase-3 and Bax/Bcl-2 [23]. In addition, another study reported that compounds extracted from AIR exhibited protective effects on neonatal rat cardiomyocytes injured by hypoxia/reoxygenation [24], further supporting its potential in ischemia–reperfusion-related injuries. Recent research demonstrated that AIRE played crucial roles in modulating oxidative stress, reducing inflammation, and enhancing neuroprotection [25, 26]. These findings collectively provide a solid foundation for exploring the effects of AIR in CIRI.

Transcriptomic and metabolomic analyses are widely applied in researching disease mechanisms and evaluating therapeutic interventions. Specifically, transcriptomics uses techniques such as RNA sequencing to investigate how diseases or pharmaceuticals influence gene expression. Metabolomics employs techniques such as mass spectrometry to analyze changes in metabolite profiles caused by diseases or medicines. These advanced techniques are crucial for unraveling the complex pathophysiological processes within the body and elucidating the underlying mechanisms of drug therapies. This study used these integrated methods to explore the mechanisms of AIRE in treating CIRI. Our findings offered new insights into the molecular mechanisms through which AIRE mediated its effects in treating CIRI.

## Results

### The identification of main chemical components in AIRE

**Fig. 1:**
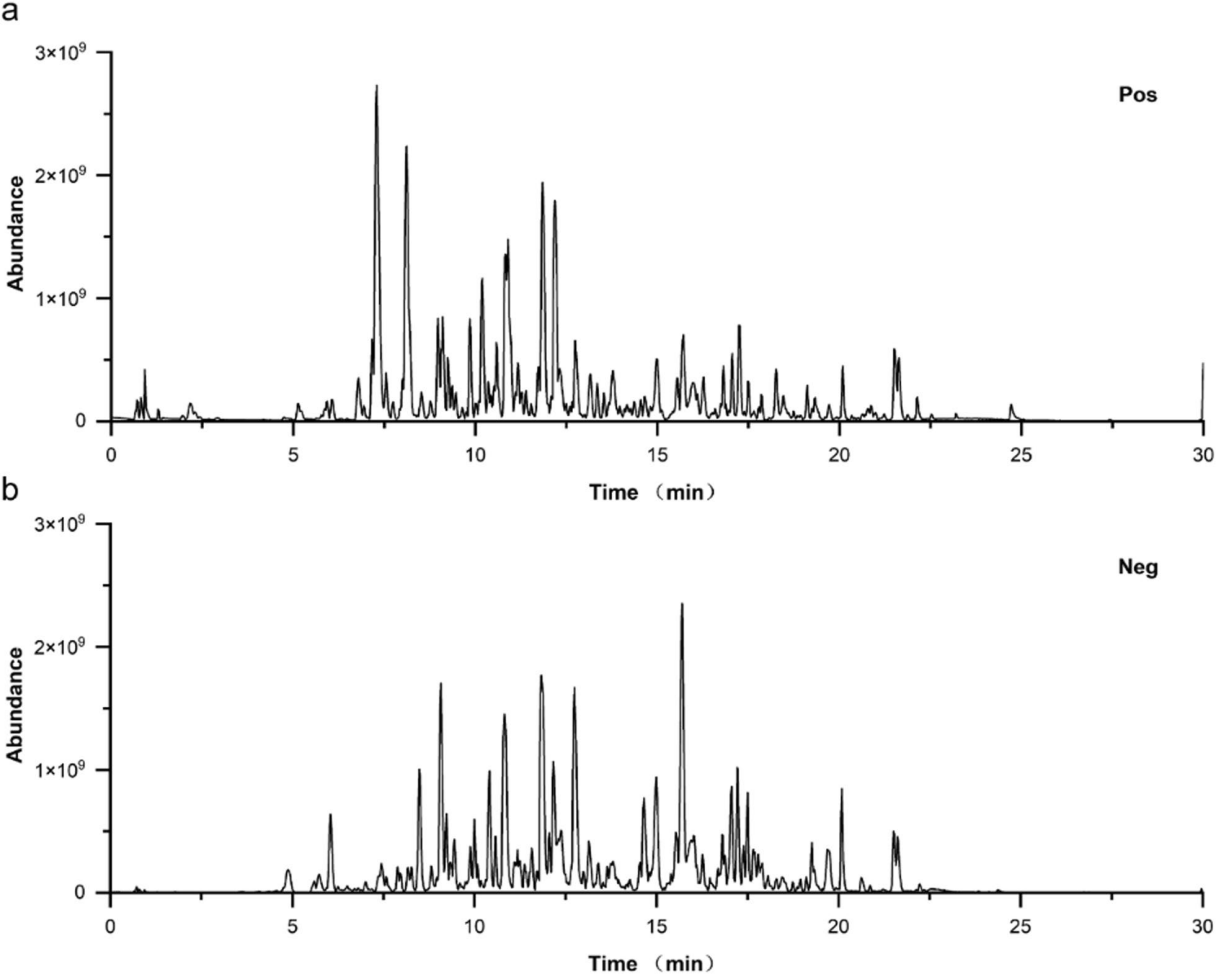
Base peak intensity chromatograms of AIRE in both positive ion mode (**a**) and negative ion mode (**b**)

The base peak intensity chromatograms of the AIRE sample were obtained using both positive and negative ion modes (Fig. 1a, b). The identified compounds in positive ion mode included Intermedine, Cinnamic acid, 1-Methoxyacetylshikonin, Acetylalkannin, Rutin, 6-Hydroxykaempferol-7-O-β-glucopyranoside, Isoquercitrin, Caffeic acid, 11-O-Acetylalkannin, Oleic acid, Ethyl oleate, Tormentic acid, EIC, and Ethyl linoleate (Table S1). The identified compounds in the negative ion mode included Chrysophanic acid, 3,4-Dimethoxycinnamic acid, Isobutylalkannin, Astragalin, Physcione, Rutin, Isoquercetin, Deoxyshikonin, Physciondiglucoside, Emodin anthrone, Angelylalkannin, Salvianolic acid B, α-Methyl-N-butyrylshikonin, β-Hydroxyisovalerylshikonin, Lithospermidin A, Aloe emodin, Emodin, Isosalvianolic acid B, Anhydroalkannin, Eicosanyl caffeate, Tormentic acid, Palmitic acid, and Ethyl stearate (Table S2).

### AIRE alleviated brain tissue injury and neuronal damage of rats subjected to CIRI

**Fig. 2:**
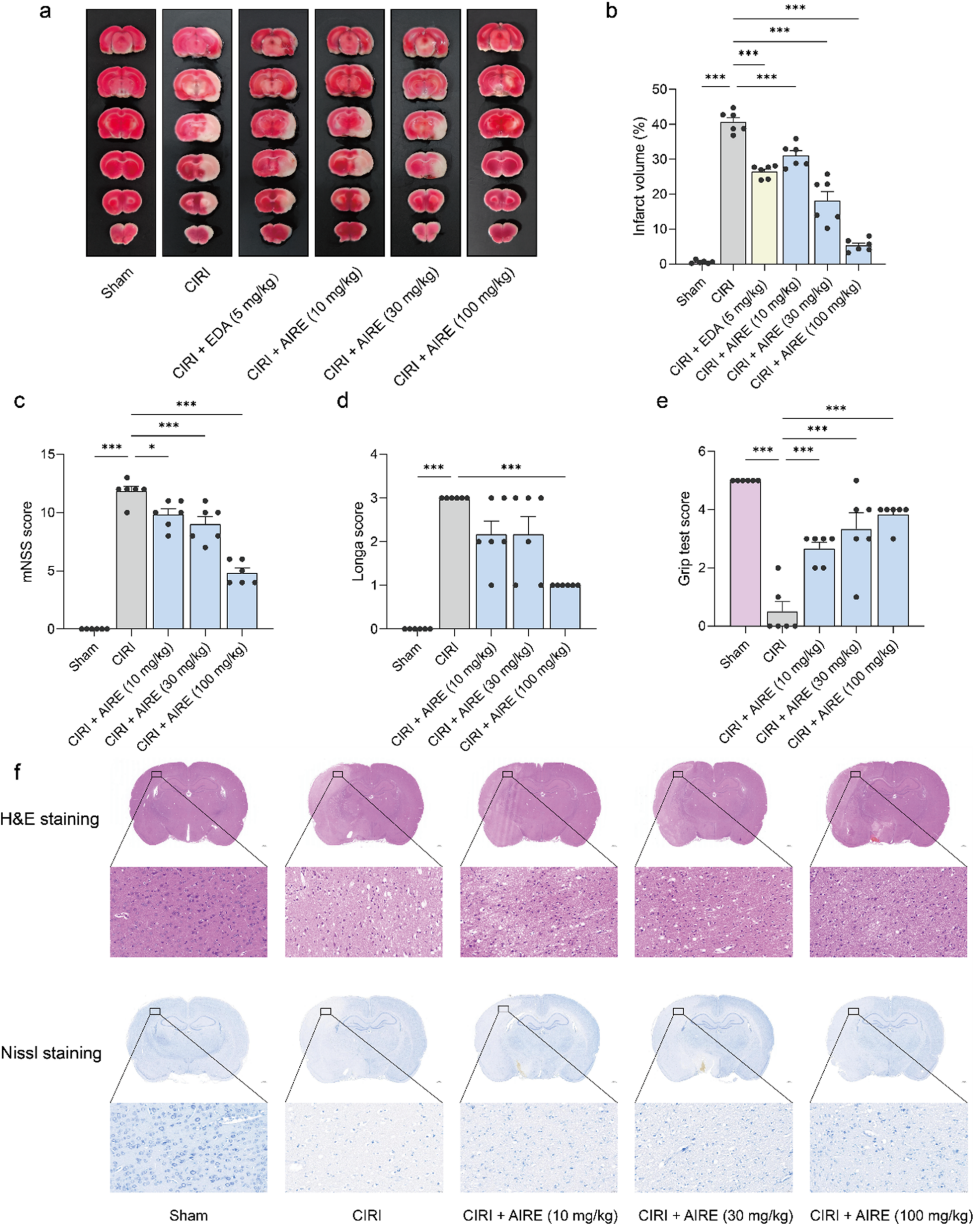
AIRE alleviated brain tissue injury and neuronal damage in rats subjected to CIRI. **a** TTC staining of brain tissue sections; **b** AIRE reduced the percentage of cerebral infarct volume caused by CIRI; **c** AIRE decreased the neurological deficit score (mNSS) induced by CIRI (n = 6); **d** AIRE decreased the neurological deficit score (Zea-Longa score) induced by CIRI (n = 6); **e** AIRE improved the grip test score after CIRI injury (n = 6); **f** H&E and Nissl staining showed that AIRE ameliorated brain tissue and neuronal damage. ^*^P < 0.05, ^**^P < 0.01, ^***^P < 0.001

At 24 h after the model was inducted, cerebral tissue damage was assessed using 2,3,5-Triphenyltetrazolium chloride (TTC) staining. The data revealed a significant increase in the volume of cerebral infarction in the CIRI model group compared to the sham group, which exhibited no infarction (Fig. 2a, b). Notably, the AIRE treatment group and the positive control edaravone (EDA) treatment group exhibited a substantial reduction in infarction volume compared to the CIRI model group, underscoring the protective effects of AIRE (Fig. 2a, b).

Additionally, impairments in neurobehavioral and motor functions were more pronounced in the CIRI group compared to the sham group, which showed no signs of impairment. Specifically, the modified neurological severity score (mNSS) and Zea-Longa scores were notably higher, and the grip strength were considerably lower in the model group, indicating more significant impairment (Fig. 2c, d, e). Conversely, in the AIRE treatment groups (10, 30, and 100 mg/kg), there was a significant amelioration in these outcomes. Both the mNSS and Zea-Longa scores decreased substantially, reflecting reduced impairment, and grip strength was also enhanced.

To investigate the therapeutic impact of AIRE on brain tissue injury following CIRI, Hematoxylin & eosin (H&E) staining and Nissl staining, were conducted to identify damages in brain tissue. It was observed that the CIRI model group exhibited significant increases in tissue cavitation and cellular morphological damage, along with a decrease in the number and staining intensity of Nissl bodies compared to the sham group (Fig. 2f). Notably, the brain tissue damages, including tissue cavitation, and cellular morphological change were alleviated in the 10, 30, and 100 mg/kg AIRE treatment groups.

### AIRE regulated metabolic profiles in brain tissue of rats subjected to CIRI

**Fig. 3:**
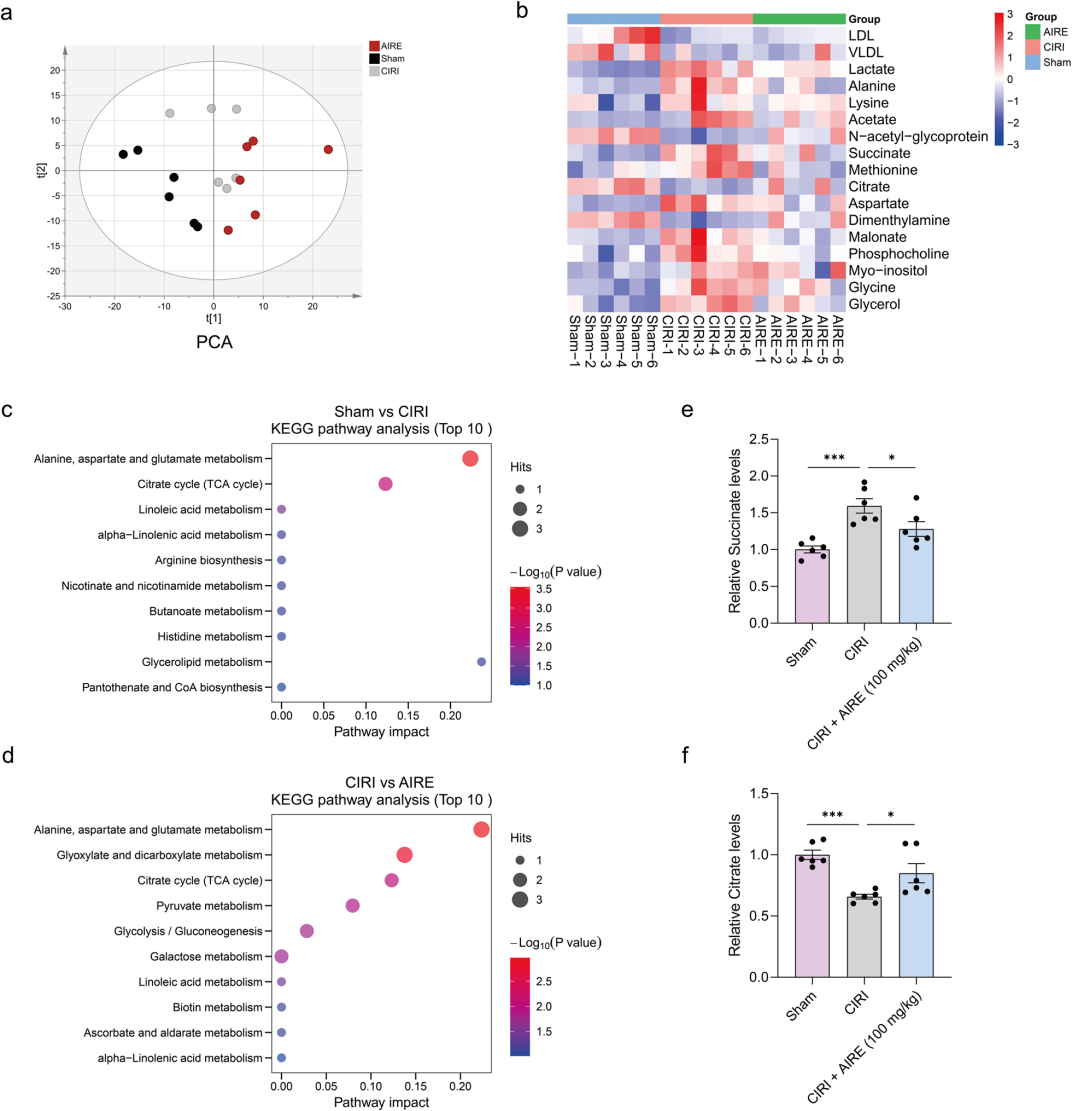
AIRE regulated metabolic profiles in brain tissue of rats subjected to CIRI. **a** PCA showed a clear distinction between the sham, model, and AIRE groups (n = 6); **b** Heatmap of differential metabolites in brain tissue; **c** A bubble plot illustrating the enrichment analysis of metabolic pathways for the differential metabolites regarding the CIRI and sham groups; **d** A bubble plot illustrating the enrichment analysis of metabolic pathways for the differential metabolites regarding the AIRE and CIRI groups; **e** The model group exhibited elevated succinate levels, but succinate levels were decreased after AIRE therapy (n = 6); **f** In the model group, citrate levels were decreased, but AIRE therapy resulted in an increase in citrate levels (n = 6). ^*^P < 0.05, ^***^P < 0.001

Principal component analysis (PCA) revealed a distinct separation among the sham group, CIRI model group, and AIRE treatment group, indicating that AIRE modulated metabolic profiles in CIRI (Fig. 3a). In comparison to the CIRI model group, the AIRE treatment group exhibited significant alterations in several differential metabolites, including N-acetyl-glycoprotein, citrate, dimethylamine, lactate, alanine, methionine, succinate, aspartic acid, and malonic acid (Fig. 3b). Subsequent metabolic pathway analysis elucidated the mechanisms underlying AIRE's effects, revealing that AIRE influenced key pathways, particularly alanine, aspartic acid, and glutamic acid metabolism, as well as the citric acid cycle (TCA cycle) (Fig. 3c, d).

Further investigation into the alanine, aspartic acid, and glutamic acid metabolism and TCA cycle pathways demonstrated that the metabolic alterations of citrate and succinate were involved in both pathways. Citrate and succinate emerged as critical metabolites within these pathways. Specifically, CIRI induced abnormal brain tissue metabolism, characterized by the downregulation of citrate and the upregulation of succinate. In comparison to the model group, AIRE treatment resulted in elevated levels of citrate and reduced levels of succinate (Fig. 3e, f). In summary, these metabolic dysfunctions in the related pathways were ameliorated in the AIRE treatment group, suggesting that AIRE might effectively regulate the disrupted energy metabolism associated with CIRI.

### AIRE regulated gene expression in brain tissue of rats subjected to CIRI

**Fig. 4:**
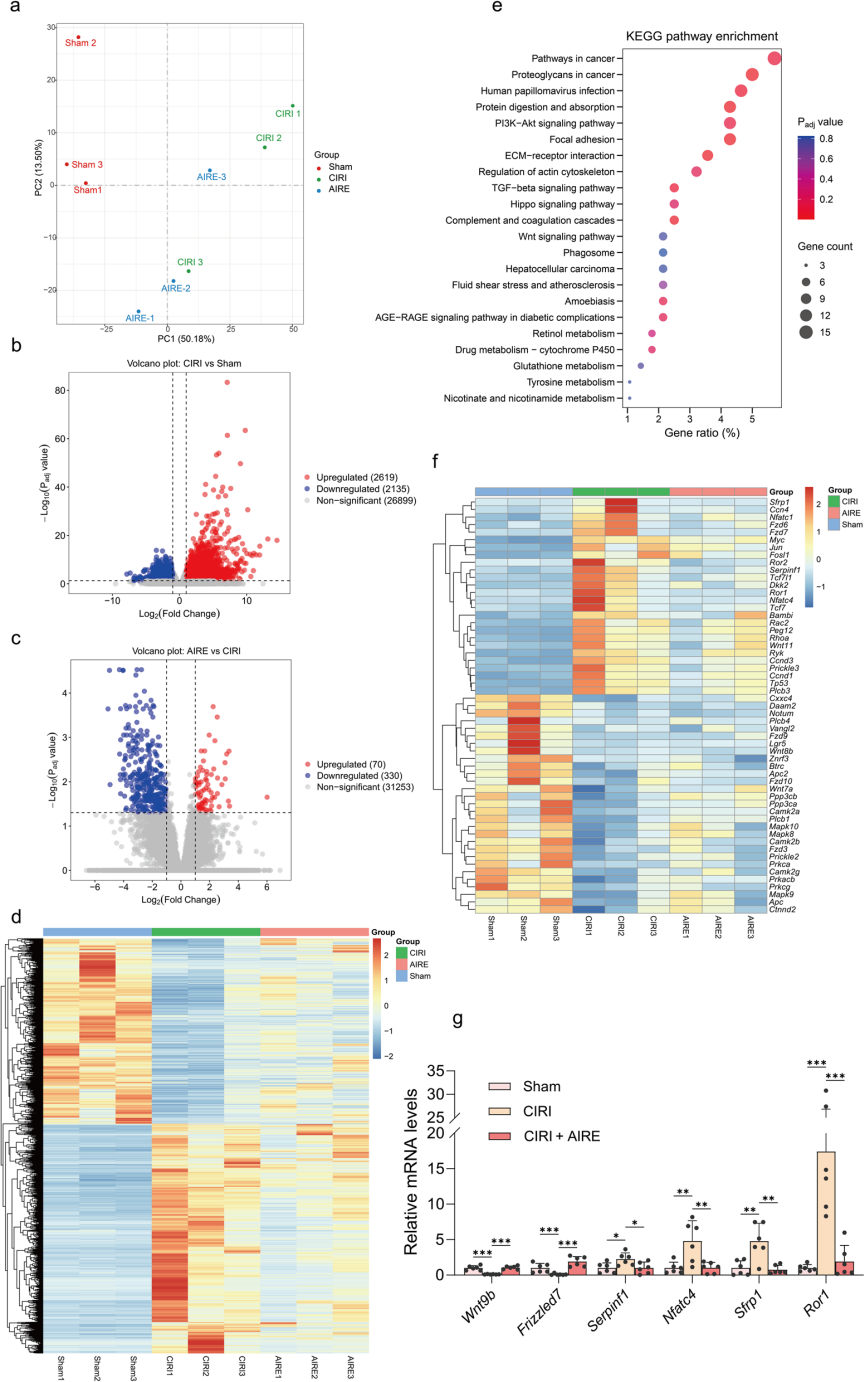
AIRE regulated gene expression in brain tissue of rats subjected to CIRI. **a** PCA analysis showed a clear distinction between the sham, model, and AIRE groups in the RNA-seq results (n = 3); **b** Volcano diagram illustrating the differential expression of genes comparing the model and sham groups; **c** Volcano diagram illustrating the differential expression of genes comparing the AIRE and model groups; **d** Heatmap of clustering analysis of all differential genes; **e** KEGG analysis on the differentially expressed genes comparing the AIRE and model groups; **f** Heatmap showing differential genes in the Wnt signaling pathway; **g** RT-qPCR validation of selected differentially expressed genes in the Wnt signaling pathway (n = 6). ^*^P < 0.05, ^**^P < 0.01, ^***^P < 0.001

RNA-seq was employed to investigate gene expression changes in CIRI following AIRE treatment. PCA results demonstrated significant separation among the gene expressions of the sham group, CIRI model group, and AIRE treatment group (Fig. 4a). Numerous genes were found to be upregulated or downregulated between different groups, as shown in the volcano plots and heatmap (Fig. 4b, c, d). Specifically, a comparison between the sham and CIRI model groups showed significant changes in 4,754 genes, with 2,619 upregulated and 2,135 downregulated (Fig. 4b). And in the AIRE treatment group, there was a significant upregulation of 70 genes and a noticeable downregulation of 330 genes compared to the CIRI model group (Fig. 4c).

KEGG pathway enrichment was conducted using the DAVID database. Notably, genes related to the TGF-β, PI3K-Akt, AGE-RAGE, and Wnt signaling pathways were found to be upregulated or downregulated in the AIRE treatment group compared to the CIRI model group (Fig. 4e). Subsequently, genes with a |Log_2_(Fold Change)| greater than 1 and *P* value less than 0.05 were examined and counted in the Wnt signaling pathway, considering all differentially expressed genes in the comparisons between the CIRI group and the sham group, as well as between the AIRE treatment group and CIRI group. It was found that 55 significantly differentially expressed genes were involved in the Wnt signaling pathway, indicating a significant involvement of this pathway in the pathophysiological process of CIRI (Fig. 4f).

Furthermore, the expression of several genes, including *Wnt9b*, *Frizzled7*, *Serpinf1*, *Nfatc4*, *Sfrp1*, and *Ror1,* was validated using RT-qPCR. Significant alterations were observed in these six key genes of the Wnt signaling pathway between the CIRI model and AIRE treatment groups (Fig. 4g). These results suggested that the regulation of Wnt signaling pathway might be a critical mechanism through which AIRE exerts its effects.

### AIRE activated the Wnt/β-catenin pathway and regulated glycolysis in brain tissue of rats subjected to CIRI

**Fig. 5:**
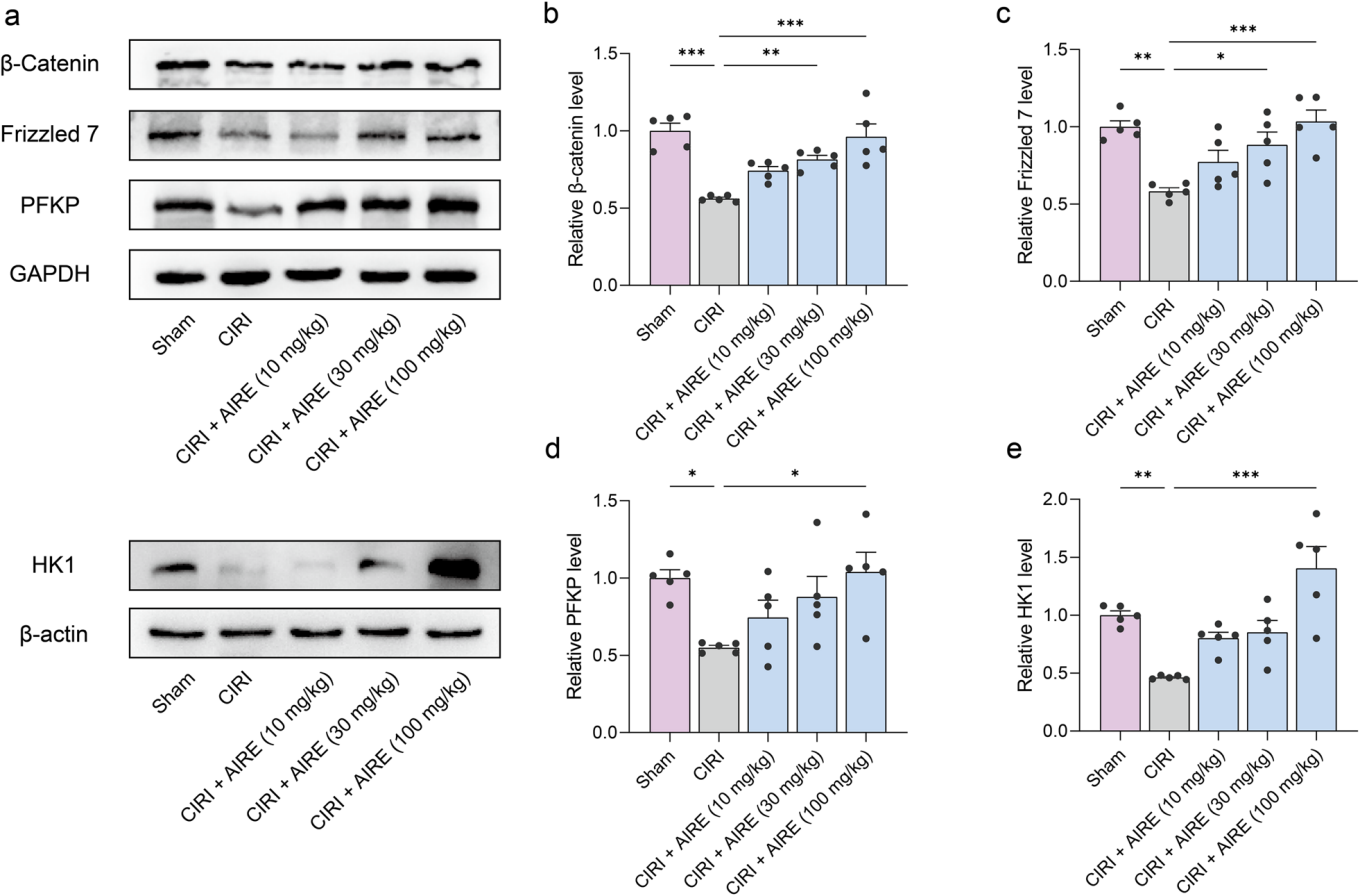
AIRE activated the Wnt/β-catenin pathway and regulated glycolysis in brain tissue of rats subjected to CIRI. **a** Western blot bands for the detected proteins (n = 5); **b** The model group exhibited a decline in the levels of β-catenin, whereas AIRE therapy increased β-catenin levels; **c** Frizzled7 levels were reduced in the model group, whereas AIRE treatment restored Frizzled7 levels; **d** The model group exhibited lower PFKP protein levels, while AIRE treatment increased the levels; **e** The expression of HK1 was decreased in the model group, but considerably elevated after AIRE therapy. ^*^P < 0.05, ^**^P < 0.01, ^***^P < 0.001

Furthermore, the western blot approach was employed to validate the alterations in key proteins within the Wnt/β-catenin pathway. The findings indicated that the levels of β-catenin and Frizzled7 in CIRI tissues were considerably lower than in the sham group. However, AIRE treatment upregulated the levels of these proteins in the CIRI model group (Fig. 5a, b, c). Since the Wnt/β-catenin pathway is crucial in glycolytic metabolism, the expression of key proteins involved in the glycolytic process, including Hexokinase 1 (HK1) and PFKP, was also examined. The results showed that CIRI led to a significant decrease in the expression of HK1 and PFKP, whereas AIRE treatment significantly upregulated these proteins (Fig. 5a, d, e). In conclusion, AIRE treatment might activate the Wnt/β-catenin route and promote glycolysis.

### AIRE regulated mitochondrial dynamics in brain tissue of rats subjected to CIRI

**Fig. 6:**
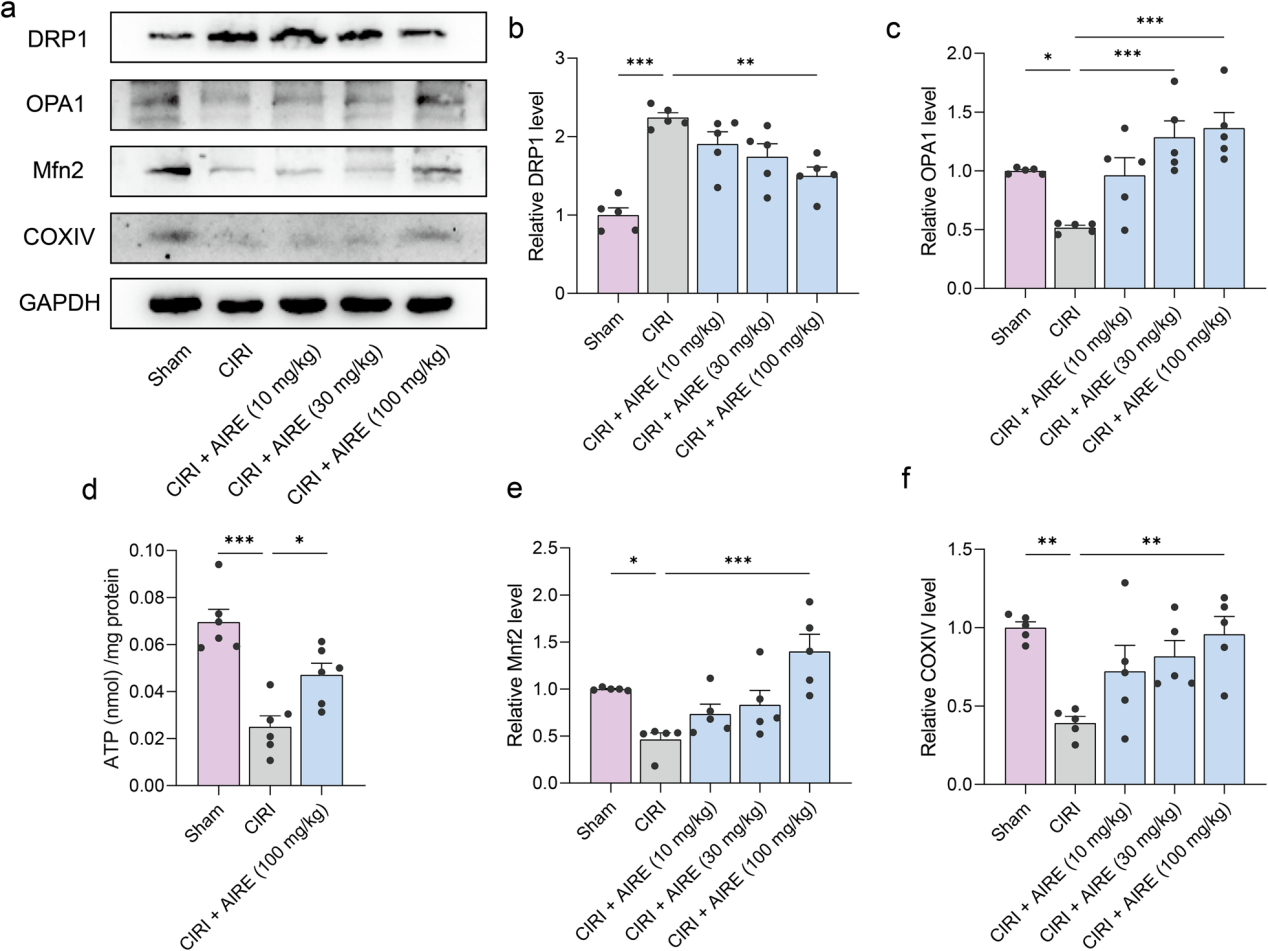
AIRE regulated mitochondrial dynamics in brain tissue of rats subjected to CIRI. **a** Western blot bands for the detected proteins (n = 5); **b** Western blot analysis revealed an elevation in DRP1 levels in the model group, but AIRE treatment decreased DRP1 levels; **c** OPA1 levels were reduced in the model group, whereas AIRE treatment increased OPA1 levels; **d** The model group exhibited lower ATP levels, while AIRE treatment (100 mg/kg) increased the ATP levels (n = 6); **e** The levels of Mnf2 was decreased in the model group, but significantly raised after AIRE therapy. **f** The model group exhibited a decrease in COXIV levels, but AIRE therapy significantly raised these levels. ^*^P < 0.05, ^**^P < 0.01, ^***^P < 0.001

Metabolomic analysis revealed that AIRE treatment interfered with several energy metabolism-related metabolites, including citrate and succinate. ATP levels were measured in the brain tissues, showing that CIRI decreased ATP levels in the injured region, whereas AIRE treatment upregulated ATP levels (Fig. 6d).

To further investigate the mechanism by which AIRE regulates energy metabolism impairment in CIRI, western blot analysis was conducted to detect key proteins related to mitochondrial dynamics. CIRI upregulated DRP1 expression and downregulated Mitofusin 2 (Mfn2) and OPA1 expression, whereas AIRE significantly downregulated DRP1 expression and upregulated Mfn2 and OPA1 expression in comparison to the model group (Fig. 6a, b, c, e). Additionally, COXIV expression was significantly lower when comparing the CIRI group to the sham group, but it was upregulated following AIRE treatment (Fig. 6f). These results suggested that AIRE improved abnormal mitochondrial division and ameliorated energy metabolism disorders.

## Discussion

A significant number of studies have indicated that herbal drugs and their preparations might help in preventing and alleviating stroke damage and reducing brain ischemia–reperfusion injury [27, 28]. These herbal drugs often contain bioactive components such as flavonoids, polyphenols, and alkaloids. Some of these components have shown potential therapeutic effects on CIRI, drawing researchers’ interest [29]. Our study demonstrated that AIRE significantly reduced cerebral infarction volume, ameliorated neurological dysfunction, and preserved brain tissue integrity and brain function in rats with CIRI. These findings indicated that AIRE might be a promising therapy for treating CIRI.

In this study, the main components of AIRE were analyzed, identifying several bioactive compounds such as Rutin, Isoquercetin, Lithospermidin A, Aloe emodin, Emodin, Isosalvianolic acid B, and Tormentic acid. Previous studies have demonstrated that these compounds possessed a range of pharmacological effects, such as anti-oxidative, anti-inflammatory, and anti-apoptotic characteristics. Some investigations demonstrated that the triterpenoids in AIRE had protective effects on cardiomyocyte damage, reversing cardiomyocyte apoptosis and autophagy following hypoxia-reoxygenation injury [30]. The flavonoids in AIRE have been found to significantly reduce infarct size in animal models of myocardial infarction and enhance cardiac function in mice [28]. These findings suggested that AIRE has a notable therapeutic impact on myocardial infarction and myocardial ischemia–reperfusion injury, implying potential therapeutic effects on ischemia or ischemia–reperfusion injury and other related disorders.

The metabolomic study underscored the significant metabolic changes induced by CIRI and highlighted the therapeutic potential of AIRE in modulating these alterations. Citrate is a crucial intermediate of the TCA cycle, essential for ATP production and cellular respiration [31]. Prior research has revealed that the level of citric acid (citrate) in the cerebrospinal fluid of rats is reduced following ischemic stroke [32]. A decrease in citrate levels suggested impaired TCA cycle activity, which could result from mitochondrial damage and reduced oxidative phosphorylation.

The accumulation of succinate during ischemia and its subsequent oxidation upon reperfusion could produce ROS, leading to oxidative stress and further mitochondrial damage [33, 34]. A previous study reported increased levels of succinate in rat serum and brain tissue following CIRI. And oxygen–glucose deprivation and reoxygenation induced primary neural stem cells to generate a substantial amount of succinate. This study found that the accumulation of succinate during ischemia reduced the activity of Cdc42 GTPase by causing Cdc42 to undergo succinylation. This, in turn, hindered the growth of neural stem cells and worsened the damage caused by CIRI [35].

This imbalance between citrate and succinate reflected disrupted energy metabolism. AIRE treatment significantly ameliorated these metabolic disturbances by elevating citrate levels and reducing succinate accumulation. This suggested that AIRE might enhance mitochondrial function and support the TCA cycle, thereby restoring efficient energy production. Normalizing citrate and succinate levels implied a protective effect for maintaining mitochondrial function.

Next, by employing RNA-seq to investigate gene expression changes in rat brain tissue, we have identified key pathways and genes modulated by AIRE treatment. Specifically, the comparison between the sham and CIRI model groups showed substantial changes in 4754 genes, with 2619 genes upregulated and 2135 downregulated. These alterations underscored the profound genetic reprogramming induced by ischemia–reperfusion injury. In the AIRE treatment group, significant modulation of gene expression was evident, with 70 genes upregulated and 330 genes downregulated compared to the CIRI model group. This differential gene expression suggested that AIRE exerted a regulatory effect, potentially restoring homeostasis disrupted by ischemic injury.

KEGG pathway enrichment analysis revealed that AIRE significantly regulated genes associated with critical signaling pathways, including the TGF-β, PI3K-Akt, AGE-RAGE, and Wnt signaling pathways. The regulation of these pathways indicated a broad-spectrum effect of AIRE on cellular signaling, which might contribute to its neuroprotective properties. Notably, 55 genes exhibiting significant differential expression were associated with the Wnt signaling pathway, highlighting its pivotal role in the pathophysiological process of CIRI and its modulation by AIRE. The validation of key genes within the Wnt pathway, such as *Wnt9b*, *Frizzled7*, *Serpinf1*, *Nfatc4*, *Sfrp1*, and *Ror1*, further supported the hypothesis that AIRE exerted its effects through the regulation of this pathway.

The Wnt/β-catenin pathway participates in multiple biological functions, such as cell metabolism, proliferation, and differentiation [36]. Our study proved that AIRE activated this pathway and regulated glycolysis in the brain tissue of rats subjected to CIRI. The verification of key protein changes in Wnt pathway revealed that β-catenin and Frizzled7 were significantly lower in CIRI tissues, while AIRE could upregulate the levels of these proteins, indicating a restoration of Wnt/β-catenin signaling activity.

Glycolysis is essential for ATP production, especially under hypoxia or ischemia conditions where mitochondrial oxidative phosphorylation is compromised [37]. Given the importance of the Wnt/β-catenin route in glycolytic metabolism [38, 39], we examined the expression of key glycolytic proteins, including HK1, and PFKP. Our results demonstrated that CIRI decreased the levels of these glycolytic enzymes, indicating impaired glycolytic flux and energy production in ischemic brain tissue. AIRE treatment, however, significantly upregulated the expression of HK1 and PFKP, suggesting a restoration of glycolytic activity. The regulation of glycolysis by AIRE through the Wnt/β-catenin route highlights a crucial aspect of its neuroprotective mechanism.

The functional state of mitochondria plays a pivotal role in determining cellular status and survival during the pathological processes of oxygen-level-dependent diseases, such as ischemic stroke and cancer [40, 41]. In this study, proteins implicated in mitochondrial dynamics were also examined. CIRI was associated with an upregulation of DRP1 and a downregulation of Mfn2 and OPA1. DRP1 is a critical mediator of mitochondrial fission [42], and its upregulation indicates enhanced mitochondrial fragmentation, often detrimental to cellular function. In contrast, Mfn2 and OPA1 are essential for mitochondrial fusion [43, 44], a process that maintains mitochondrial integrity and function. AIRE treatment significantly downregulated DRP1 expression while upregulating Mfn2 and OPA1, suggesting a shift towards improved mitochondrial fusion and reduced fragmentation. This modulation of mitochondrial dynamics is crucial, as balanced fission and fusion are essential for maintaining mitochondrial health, optimizing energy production, and preventing apoptosis. Additionally, the expression of COXIV, necessary for mitochondrial respiratory capacity [45], was downregulated in the CIRI group but was upregulated following AIRE treatment, further indicating enhanced mitochondrial function and energy metabolism.

The primary limitation of this study was mainly related to the RNA-seq pathway enrichment analysis, where we identified a number of enriched pathways. However, it was not feasible to investigate all these pathways within the scope of our research. For example, many differentially expressed genes are related to the TGF-β, PI3K-Akt, and AGE-RAGE signaling pathways, which could also be involved in the mechanisms through which AIRE exerted its effects. We ultimately focused on the Wnt signaling pathway and confirmed its key role, demonstrating that AIRE alleviated CIRI, at least in part, through the Wnt/β-catenin pathway. It was important to note that AIRE's effects might involve other pathways as well, which we plan to examine in future research.

## Conclusions

**Fig. 7:**
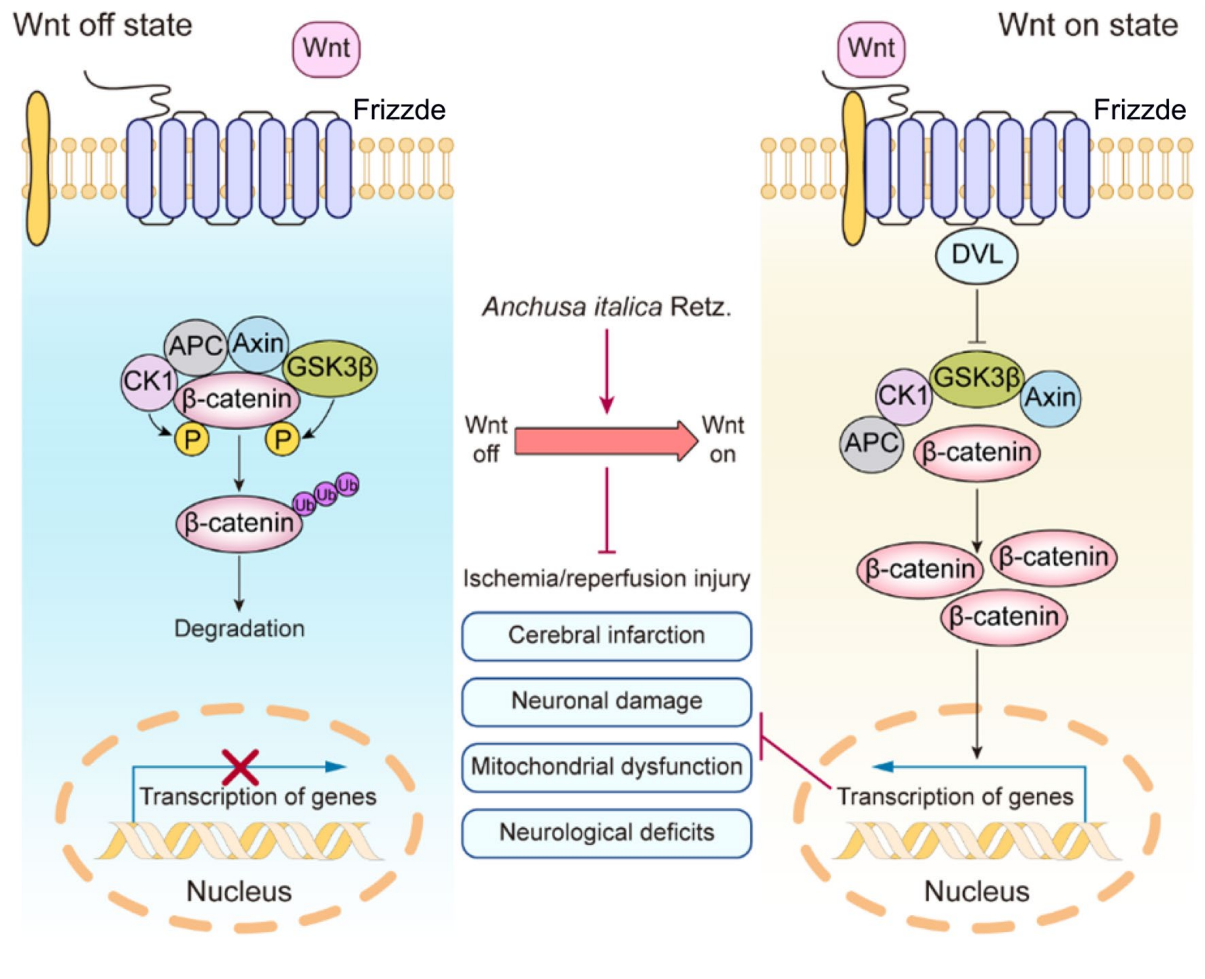
Mechanism of AIRE in alleviating cerebral ischemia–reperfusion injury through activation of the Wnt/β-catenin pathway

This study revealed that AIRE contained various bioactive components and significantly ameliorated CIRI. In animal experiments, AIRE markedly decreased the size of cerebral infarction and alleviated neurological dysfunction in CIRI rats. The underlying mechanism might have involved the regulation of mitochondrial dysfunction induced by CIRI through the Wnt/β-catenin signaling pathway (Fig. 7), thereby inhibiting mitochondrial energy disturbances and affecting the TCA cycle, as well as alanine, aspartic acid, and glutamate metabolism. These findings might provide a new approach to preventing and treating CIRI by utilizing AIRE.

## Experimental section

### AIRE preparation

AIR was obtained from Xin Jiang Yao Du Pharmaceutical Co., LTD (Xinjiang, China, #20190110). The steps for extracting AIRE from AIR have been described in our previous study [23]. Briefly, the dried and ground AIR plant was first soaked in a solution of 60% v/v aqueous ethanol for 6 h. Afterwards, it was extracted by utilizing a 10 L reflux reactor at 90 °C for 2 h, with a liquid–solid ratio of 1:6, using 75% v/v aqueous ethanol as the extracting agent. Following two extraction cycles, the whole extract was concentrated with reduced pressure at 60 °C. This process resulted in a viscous extract with a concentration of about 30 mg/mL. The extract was subsequently purified through a D101 macroporous resin column, using 70% ethanol as the eluent. Finally, after the ethanol was removed, the eluent was vacuum freeze-dried.

### Analysis of the main components of AIRE

To analyze of the chemical components of AIRE, the samples were prepared and subjected to chromatographic and mass spectrometric conditions. For sample preparation, the sample was mixed with 80% methanol and ultrasonicated for 30 min, with a liquid–solid ratio of 40:1. Then, the suspension was centrifuged at 4 °C and 12,000 rpm for 10 min. Subsequently, the supernatant was pipetted into an injection vial for analysis.

The Vanquish Flex UHPLC system, equipped with an ACQUITY UPLC HSS T3 column (2.1 mm × 100 mm, 1.7 μm), was used for chromatographic analysis. The mobile phase comprised an aqueous solution containing 0.1% formic acid (phase A) and acetonitrile (phase B) flowing at a rate of 0.3 mL/min. The column temperature was kept constant at 40 °C. Table 1 displayed the elution gradient.

**Table 1:** Elution gradient

Time (min)	Mobile phase	
	A (v%)	B (v%)
0	98	2
1.0	98	2
14.0	70	30
25.0	0	100
28.0	0	100
28.1	98	2
30.0	98	2

The Q Exactive hybrid quadrupole-orbitrap mass spectrometer (Thermo Fisher Scientific) with a HESI-II spray probe was used to obtain mass spectrometric data. The parameters were set as follows: positive ion source voltage at 3.7 kV, negative ion source voltage at 3.5 kV, heated capillary temperature at 320 °C, sheath gas pressure at 30 psi, auxiliary gas pressure at 10 psi, and desolvation temperature at 300 °C. Nitrogen was employed as the sheath and auxiliary gas, as well as the collision gas. Data acquisition was performed in "Full scan/dd-MS^2^" mode. The full scan parameters were set to a resolution of 70000, an auto gain control target of 1 × 10^6^, and a maximum isolation time of 50 ms. The dd-MS^2^ data were collected with a resolution of 17500, an auto gain control target of 1 × 10^5^, a maximum isolation time of 50 ms, a loop count of the top 10 peaks, an isolation window of m/z 2, collision energies of 10, 30, and 60 V, and an intensity threshold of 1 × 10^5^.

Data analysis was conducted using Progenesis QI 3.0 software (Waters Corp., MA, USA), which included steps for raw data introduction, peak extraction, and adduct deconvolution. Identification was determined based on the mass error of the parent ion, match degree of fragment ions, isotope distribution, and peak area by searching against a theoretical database constructed from literature and public databases.

### Animals

Adult male Sprague–Dawley (SD) rats (SPF grade, weight: 240–280 g) were provided by Beijing HFK Bioscience Co., Ltd. (Beijing, China, Certificate No: SCXK (Beijing) 2019–0008). All experiments were conducted in accordance with protocols approved by the Ethics Committee of the Institute of Materia Medica, Chinese Academy of Medical Sciences, and Peking Union Medical College. All SD rats underwent a 7-day adaptation period to acclimate to laboratory conditions before being randomly assigned to various groups based on experimental requirements.

### Establishment of CIRI model and animal grouping

The CIRI model, as outlined in prior work [46], was utilized with slight adjustments. Rats were anesthetized using 2.5% isoflurane, maintained at 1.5%, and positioned supine. After sterilizing the surgical area, an incision was performed to uncover the common carotid artery (CCA) through blunt dissection from the surrounding tissues. Both the external carotid artery (ECA) and internal carotid artery (ICA) were carefully isolated and exposed. Initially, vascular clips were used to occlude the ICA and CCA. The ECA was then incised approximately 0.5 cm from its bifurcation with the ICA. Following this, a nylon thread was inserted through ECA into the ICA. Afterwards, the vascular clip on the ICA was taken off, allowing the thread to be transferred forward into the middle cerebral artery (MCA). Then, the vascular clip on the CCA was also removed. After 1.5 h of induced ischemia, the thread was removed to initiate the restoration of blood supply. Rats in the sham group received the same surgical procedures, excluding the insertion of a nylon thread.

Besides the sham group, animals that underwent successful operations were randomly allocated into the CIRI model group, the treatment groups receiving 10, 30, and 100 mg/kg AIRE, and the positive control EDA (Simcere, China, #H20031342) group, respectively.

### Evaluation of neurological function

Neurological impairment was assessed using the mNSS, Zea-Longa rating, and grip strength test at 24 h post-ischemia–reperfusion. All neurological assessments were carried out by researchers unaware of the experimental assignments. The mNSS test was evaluated using a range from 0 to 18 and consisted of assessments for motor function, sensory perception, reflexes, and balance. A score of 0 indicated normal condition, while a score of 18 indicated the most severe deficiency. Severe CIRI model rats with scores of 10 to 15 were adopted for this study.

### Measurement of cerebral infarct volume in TTC staining

Deep anesthesia of rats was induced using 2.5% isoflurane. The cerebrum was then harvested and sectioned coronally into 2 mm slices. These slices were then incubated in a 1% solution of TTC for 30 min. Following incubation, the tissues were fixed in 4% formaldehyde and subsequently photographed. The cerebral infarct volume was quantified using ImageJ software (National Institutes of Health, USA). The percentage of cerebral infarct volume was calculated using the formula: [total cerebral infarct volume—(ipsilateral hemisphere volume—contralateral hemisphere volume)] / contralateral hemisphere volume × 100%.

### H&E staining and Nissl staining

Brain damage induced by CIRI was investigated using both H&E staining and Nissl staining. Brain tissue samples, preserved in 4% formaldehyde solution, were encased in paraffin and sliced into 5 μm thin sections. These slices were then used to conduct H&E and Nissl staining to observe tissue or neuronal damage. The stained slices were subsequently scanned using a Pannoramic MIDI scanner (3DHISTECH Ltd., Hungary).

### Metabolomic analysis

Metabolomic analysis was conducted on penumbra brain tissues from the sham group, the model group and the 100 mg/kg treatment group. Tissue samples were homogenized at 15 mg per 1.5 mL in ice-cold lysis/extraction buffer (chloroform: methanol: water at a 2:2:1 ratio). After centrifugation at 12,000 rpm for 15 min at 4 °C, the supernatant from each sample was collected for analysis. The ^1^H-NMR spectra were acquired using a Bruker 600 MHz Avance III NMR spectrometer and analyzed with MestReNova software (Mestrelab Research). Then signal peaks in the spectra were matched to specific metabolites using the documented literature as well as the NMR database. Finally, the different metabolites were selected to investigate metabolic pathways by MetaboAnalyst 6.0 [47].

### Transcriptomic analysis

From each of the sham group, the CIRI model group, and the 100 mg/kg treatment group, three rats were randomly selected for further study. Initially, total RNA was acquired from the penumbra brain tissues with RNAiso Plus (NovBio, China, #9109), and its purity was enhanced with AMPure XP beads. The RNA quality was subsequently evaluated using an Agilent 2100 Bioanalyzer. Subsequent steps involved generating RNA-seq libraries utilizing the TruSeq RNA Sample Prep Kit (Illumina, USA), followed by sequencing on an Illumina HiSeq2500 system. To standardize gene expression levels, all data were normalized using the fragments per kilobase per million (FPKM) value. Genes exhibiting a |Log_2_(Fold Change)| more than 1 and *P* value less than 0.05 were considered to be significantly differentially expressed. Pathways associated with identified DEGs were enriched by KEGG, using the DAVID database [48].

### Reverse transcription-quantitative PCR (RT-qPCR)

Samples obtained from the penumbra region of the sham group, CIRI model group, and 100 mg/kg treatment group were homogenized to extract total RNA using RNAiso Plus (NovBio, China, #9109). cDNA was generated with the HiScript III All-in-one RT SuperMix Perfect for qPCR (Vazyme Biotech). Real-time PCR was conducted on the ABI PRISM 7900HT Sequence Detection System (Applied Biosystems). The primers are shown in Table 2**.**

**Table 2:** The primer list

Name	Forward (5’-3’)	Reverse (5’–3’)
*Wnt9b*	CGCGAGGAGATGCAAGAGT	GTCAGGACCTCACGACCG
*Frizzde7*	GAGGATACCGCTCTGTCTGC	TCCGGGAGGAGGACTGTAAG
*Nfatc4*	GGGCGTCTCAGAGAAAAGGA	TCCCCGAACACCAGCTTAAA
*Ror1*	CTTCCAGGAGACCACGCTTT	GGGGGTTGCTGAGGTTACTC
*Sfrp1*	ATCGGAGGCCATCATCGAAC	ACAGTCGGCGCCATTCTTTA
*Serpinf1*	AATTGCCCGGTCTACAAGGG	GTCCTGTCCTCGTCCAAGTG
*β-actin*	TGAGCTGCGTTTTACACCCT	GCCTTCACCGTTCCAGTTTT

### ATP assay

Tissue samples from the penumbra region were homogenized in ice-cold lysis/extraction buffer at 30 Hz for 30 min. Following centrifugation at 4 °C (12000 rpm for 15 min), the supernatant from each sample was gathered. ATP levels were quantified using an ATP test kit (Beyotime, China, #S0026) according to the manufacturer’s instructions.

### Western blot analysis

The penumbra tissues were homogenized in RIPA buffer with added protease and phosphatase inhibitors. Total protein was assessed using a BCA protein assay kit (Applygen, China, #P1511). The proteins were separated by SDS-PAGE and transferred onto PVDF membranes. The membranes were then incubated with primary antibodies from Abcam: anti-DRP1 antibody (1:1000, #ab184247), anti-Mfn2 antibody (1:1000, #ab124773), anti-OPA1 antibody (1:1000, #ab157457), anti-HK1 antibody (1:1000, #ab150423), anti-PFKP antibody (1:2000, #ab204131), anti-Frizzled 7 antibody (1:1000, #ab64636), anti-beta Catenin antibody (1:5000, #ab32572), anti-COX IV antibody (1:2000, #ab16056), anti-Actin antibody (1:20000, #ab198991), and anti-GAPDH antibody (1:20000, #ab128915). The membranes were subsequently treated with secondary antibodies (goat anti-rabbit, Abcam, #ab205718; goat anti-mouse, Abcam, #ab205719) for a duration of 2 h at room temperature. Bands were recognized using an enhanced chemiluminescence system.

### Statistical analysis

The experimental data are displayed as mean ± SEM and processed with GraphPad Prism 7.05. For normally distributed data, statistical significance was evaluated with one-way ANOVA, followed by Tukey's multiple comparisons test. In cases of unequal standard deviations, the Brown-Forsythe and Welch ANOVA tests were applied. For non-normally distributed data, the Kruskal–Wallis test was utilized. A *P* value below 0.05 was deemed statistically significant.

## Supplementary Information

Supplementary material 1: Table S1. Identification results of compounds detected in positive ion mode, Table S2. Identification results of compounds detected in negative ion mode.

## Data Availability

The datasets generated during and/or analyzed during the current study are available from the corresponding authors on reasonable request.
