# Supplementary material for: Metabolomic and transcriptomic analyses revealed potential mechanisms of Anchusa italica Retz. in alleviating cerebral ischemia–reperfusion injury via Wnt/β-catenin pathway modulation
Source: Nat Prod Bioprospect. 2025 Jan 8;15(1):11. doi: 10.1007/s13659-024-00495-3 (PMC11711721; doi:10.1007/s13659-024-00495-3)
Supplement: Supplementary file 1 — Supplementary material 1: Table S1. Identification results of compounds detected in positive ion mode, Table S2. Identification results of compounds detected in negative ion mode. [file 13659_2024_495_MOESM1_ESM.docx]

**Table S****1** Identification results of compounds detected in positive ion mode

| No. | Compound | m/z | Retention time (min) | Adducts | Formula | Score | Fragmentation Score | Mass Error (ppm) |
| --- | --- | --- | --- | --- | --- | --- | --- | --- |
| 1 | Intermedine | 300.1797 | 6.04 | M+H | C_15_H_25_NO_5_ | 49.70 | 54.30 | -2.74 |
| 2 | Cinnamic Acid | 131.0490 | 9.77 | M+H-H_2_O | C_9_H_8_O_2_ | 51.20 | 57.40 | -0.66 |
| 3 | Astragalin | 449.1067 | 9.92 | M+H | C_21_H_20_O_11_ | 49.60 | 52.90 | -2.60 |
| 4 | 1-Methoxyacetylshikonin | 345.1328 | 10.26 | M+H | C_19_H_20_O_6_ | 42.50 | 40.90 | -1.29 |
| 5 | Acetylalkannin | 330.1086 | 10.58 | M+ | C_18_H_18_O_6_ | 47.30 | 44.00 | -3.77 |
| 6 | Rutin | 611.1587 | 10.79 | M+Na, 2M+H, M+H | C_27_H_30_O_16_ | 52.00 | 64.20 | -3.27 |
| 7 | 6-Hydroxykaempferol -7-O-beta-glucopyranoside | 465.1013 | 10.81 | M+H | C_21_H_20_O_12_ | 51.40 | 61.80 | -3.03 |
| 8 | Isoquercetin | 465.1017 | 11.13 | M+H | C_21_H_20_O_12_ | 52.70 | 67.40 | -2.32 |
| 9 | Caffeic Acid | 163.0385 | 12.73 | M+H-2H_2_O, M+H-H_2_O, M+H | C_9_H_8_O_4_ | 47.80 | 42.70 | -2.64 |
| 10 | 11-O-Acetylalkannin | 330.1086 | 12.75 | M+ | C_18_H_18_O_6_ | 47.00 | 43.60 | -3.72 |
| 11 | Oleic Acid | 305.2464 | 16.29 | M+Na | C_18_H_34_O_2_ | 48.60 | 50.70 | 4.66 |
| 12 | Ethyl oleate | 293.2826 | 17.12 | M+H-H_2_O, M+H-2H_2_O | C_20_H_38_O_2_ | 46.70 | 42.00 | -4.10 |
| 13 | Tormentic acid | 489.3554 | 20.64 | M+H | C_30_H_48_O_5_ | 50.20 | 57.90 | -4.15 |
| 14 | EIC | 303.2305 | 21.50 | M+Na | C_18_H_32_O_2_ | 46.80 | 40.70 | 3.61 |
| 15 | Ethyl Linoleate | 273.2563 | 23.13 | M+H-2H_2_O | C_20_H_36_O_2_ | 51.30 | 65.30 | -4.35 |

**Table S2** Identification results of compounds detected in negative ion mode

| No. | Compound | m/z | Retention time (min) | Adducts | Formula | Score | Fragmentation Score | Mass Error (ppm) |
| --- | --- | --- | --- | --- | --- | --- | --- | --- |
| 1 | Chrysophanic acid | 299.0566 | 8.52 | M+FA-H | C_15_H_10_O_4_ | 55.50 | 83.90 | 1.73 |
| 2 | 3,4-Dimethoxycinnamic acid | 207.0655 | 8.90 | M-H | C_11_H_12_O_4_ | 46.80 | 44.30 | -3.93 |
| 3 | Isobutylalkannin | 357.1350 | 9.75 | M-H | C_20_H_22_O_6_ | 48.70 | 51.00 | 1.67 |
| 4 | Astragalin | 447.0941 | 9.91 | M-H | C_21_H_20_O_11_ | 56.30 | 85.30 | 1.80 |
| 5 | Physcione | 329.0674 | 10.24 | M+FA-H | C_16_H_12_O_5_ | 51.30 | 62.30 | 2.66 |
| 6 | Rutin | 609.1474 | 10.83 | M-H | C_27_H_30_O_16_ | 53.70 | 71.30 | 2.06 |
| 7 | Isoquercetin | 463.0894 | 11.16 | M-H | C_21_H_20_O_12_ | 48.20 | 46.40 | 2.53 |
| 8 | Deoxyshikonin | 317.1035 | 11.25 | M+FA-H | C_16_H_16_O_4_ | 50.20 | 57.50 | 1.79 |
| 9 | Physciondiglucoside | 653.1741 | 11.29 | M+FA-H | C_28_H_32_O_15_ | 48.80 | 50.20 | 2.85 |
| 10 | Emodin anthrone | 301.0722 | 12.08 | M+FA-H | C_15_H_12_O_4_ | 47.20 | 42.20 | 1.77 |
| 11 | Angelylalkannin | 369.1348 | 12.53 | M-H | C_21_H_22_O_6_ | 48.70 | 49.40 | 1.18 |
| 12 | Salvianolic acid B | 717.1484 | 12.74 | M-H | C_36_H_30_O_16_ | 50.10 | 63.70 | 3.13 |
| 13 | α-methyl-n-butyrylshikonin | 417.1559 | 12.74 | M+FA-H | C_21_H_24_O_6_ | 47.10 | 58.10 | 1.09 |
| 14 | β-Hydroxyisovalerylshikonin | 433.1509 | 13.71 | M+FA-H | C_21_H_24_O_7_ | 50.50 | 55.50 | 1.24 |
| 15 | Lithospermidin A | 433.1512 | 13.90 | M+FA-H | C_21_H_24_O_7_ | 54.40 | 76.10 | 2.04 |
| 16 | Aloe emodin | 269.0462 | 15.19 | M-H | C_15_H_10_O_5_ | 47.40 | 43.80 | 2.43 |
| 17 | Emodin | 315.0517 | 15.44 | M+FA-H | C_15_H_10_O_5_ | 49.20 | 51.50 | 2.61 |
| 18 | Isosalvianolic acid B | 717.1486 | 16.51 | M-H | C_36_H_30_O_16_ | 46.50 | 40.60 | 3.50 |
| 19 | Anhydroalkannin | 269.0824 | 17.21 | M-H | C_16_H_14_O_4_ | 47.60 | 44.70 | 1.73 |
| 20 | Eicosanyl caffeate | 505.3552 | 18.87 | M+FA-H | C_29_H_48_O_4_ | 49.90 | 57.00 | 3.70 |
| 21 | Tormentic acid | 487.3438 | 20.62 | M-H, M+FA-H | C_30_H_48_O_5_ | 52.70 | 66.90 | 1.84 |
| 22 | Palmitic Acid | 301.2390 | 20.78 | M+FA-H | C_16_H_32_O_2_ | 48.40 | 47.60 | 2.14 |
| 23 | Ethyl stearate | 343.2858 | 21.94 | M+FA-H | C_19_H_38_O_2_ | 54.40 | 75.90 | 1.56 |
